# Population structure and genetic differentiation of tea green leafhopper, *Empoasca* (*Matsumurasca*) *onukii*, in China based on microsatellite markers

**DOI:** 10.1038/s41598-018-37881-0

**Published:** 2019-02-04

**Authors:** Li Zhang, Fuping Wang, Li Qiao, Christopher H. Dietrich, Masaya Matsumura, Daozheng Qin

**Affiliations:** 1 Key Laboratory of Plant Protection Resources and Pest Management of the Ministry of Education, Entomological Museum, Northwest A&F University, Yangling, Shaanxi 712100 China; 2 Yangling Xianglin Agricultural Science & Technology Chemical Company Limited, Yangling, Shaanxi 712100 China; 3 College of Agronomy, Xinyang Agricultural and Forestry University, Xinyang, Henan 464000 China; 4 Illinois Natural History Survey, Prairie Research Institute, University of Illinois, 1816 S. Oak Street, Champaign, IL 61820 USA; 5 Department of Planning and Coordination, Headquarters, National Agriculture and Food Research Organization, 3-1-1 Kannondai, Tsukuba, Ibaraki 305-8517 Japan

## Abstract

The tea green leafhopper, *Empoasca* (*Matsumurasca*) *onukii* Matsuda, is one of the dominant pests in major tea production regions of East Asia. Recent morphological studies have revealed variation in the male genitalic structures within and among populations. However, the genetic structure of this pest remains poorly understood. This study explores the genetic diversity and population structure of this pest in nineteen populations from the four main Chinese tea production areas using microsatellite markers, with one Japanese population also examined. The results show low to moderate levels of genetic differentiation with populations grouped into four clusters, i.e. the Jiangbei group, the Southwest group 1, the Southwest group 2 and the South China group. Populations from China have a close phylogenetic relationship but show significant isolation by distance. Lower genetic diversity and genetic differentiation of *E*. (*M*.) *onukii* were found in the Kagoshima population of Japan. Evidence for genetic bottlenecks was detected in the South China and Jiangnan populations. Population expansion was found in the Southwest, Jiangbei and Kagoshima populations. This is the most extensive study of the population genetics of this species and contributes to our understanding of its origin and evolutionary history.

## Introduction

The tea green leafhopper is one of the most dominant pests in the major tea production regions of East Asia^1,2^. It was first described in 1952 as an *Empoasca* leafhopper injurious to tea shrubs in Japan^3^. Although considerable economic losses caused by this pest have drawn substantial attention by farmers and government bodies in China since the 1950s, two incorrect scientific names, *Empoasca* (s. str.) *vitis* (Goëthe) and *Jacobiasca formosana* (Paoli) continued to be applied to the pest in mainland China and Taiwan, respectively, in both basic and applied research for many years^4–7^. Recent morphological and molecular evidences reveal this pest in China is the same species that occurs on tea in Japan^1,8^. Thus, the correct scientific name of the tea green leafhopper in China has been established as *Empoasca* (*Matsumurasca*) *onukii* Matsuda^1^. Damage caused by adults and nymphs of this pest has been described as the classical symptoms of hopperburn in young tea leaves and shoots. This damage results from the leafhoppers’ piercing-sucking mode of feeding^9^, leading to yield losses averaging 10–50% in China^10,11^ and up to 33% in Japan^12^.

Despite previous studies reporting that tea production originated in Southeast China more than three thousand years ago^13^, tea trees are now widely cultivated in more than 34 countries across Asia, Africa, Latin America, and Oceania^14^. However, so far, this pest has been officially reported only from three countries (Japan, Vietnam and China) in Asia^1,2,15^. In China, tea plantations now account for half the overall tea production by area worldwide, and comprise four tea regions: Jiangbei (North Central), Jiangnan (South Central), South China and Southwest China. These regions are designated based on the ecological conditions, the history of tea production, and the distribution and cultivation characteristics of tea varieties^16,17^.

Recent morphological study has revealed variation in the structure of the male genitalia (aedeagus) within and among populations of the tea green leafhopper^1,18^. Thus, it is reasonable to speculate that some genetic differentiation has developed, possibly as a result of restriction of gene flow among populations by geographic barriers (e.g., large rivers and mountains) between these four Chinese tea regions. In Japan, the predominant tea cultivar ‘Yabukita’, is cultivated intensively in Shizuoka Prefecture in southern Honshu and Kagoshima Prefecture in Kyoto^19^, both well separated from the Chinese tea production areas, leading us to expect the genetic characteristics of Japanese *E*. (*M*.) *onukii* to be different from the Chinese populations.

Previous attempts to explore the genetic structure of this pest include one study that compared samples from a small number of Chinese tea gardens using the RAPD method^20^ and another study that developed a haplotype network based on several geographic populations using mtDNA *COI* and 16S rRNA sequence data^21–25^. However, specimen sampling methods in these studies were not consistent, nor were the male genitalic characters studied to confirm the species identification and population-level variation. Unfortunately, given the past confusion over the identity of the species, it is possible that samples used in previous genetic studies have included other species besides *E*. (*M*.) *onukii*, and this could lead to a misunderstanding of Chinese *E*. (*M*.) *onukii* genetic structure. Because almost all *Empoasca* species (>200 species occur in the Chinese fauna) can be positively identified only by examining the male genitalia and multiple *Empoasca* species may coexist in the same habitat, it is crucial to confirm the identity of each individual specimen morphologically before it is incorporated into any population genetics study^1–3^.

Recently, 1569 microsatellite loci including 87 different repeat units have been found in the genome of the tea green leafhopper, among which triplet repeats (27.84%) and dinucleotide repeats (70.26%) were the most abundant^26^. Twenty-one microsatellite markers were previously developed and selected for genetic studies of *E*. (*M*.) *onukii* in Chinese tea production areas^27^. Here we report the results of a comprehensive population genetic study based on these previously identified markers, incorporating specimens confirmed as *E*. (*M*.) *onukii* based on examination of male genitalia and obtained from all major Chinese tea production areas. Similar analyses have been conducted previously to explore the genetic differentiation, population structure and population dynamics of other species of Hemiptera^28–36^.

The purpose of this study was to (i) assess the genetic differentiation and population dynamics of *E*. (*M*.) *onukii* populations through comparative studies of populations in four tea production regions; (ii) examine effects of geographic isolation on the genetic differentiation and observed population genetic patterns of the tea green leafhopper; and (iii) elucidate the origin and evolutionary history of the species. This study represents the most extensive use of microsatellite markers to elucidate the origin and evolutionary history of *E*. (*M*.) *onukii*.

## Results

### Genetic diversity

*Eo*-9 exhibited a significant excess of homozygosity. More than a 50% amplified fragment dropout was found for *Eo*-1-65 and *Eo*-9. *Eo*-29, *Eo*-37, *Eo*-54, *Eo*-1-52, *Eo*-1-57, *Eo*-83, *Eo*-1-5, *Eo*-F-8 and *Eo*-1-77 deviated from Hardy-Weinberg equilibrium (pHWE < 0.01) across different populations because of null alleles. However, null allele frequencies, lower than 0.2 for 18 markers, yielded little effect on genetic diversity and population structure, excluding *Eo*-4-5 (null allele frequency > 0.2 in all populations)^37,38^. The average frequency of null alleles at 18 markers over the populations ranged from 0.027 to 0.075 (Table 1). So, excluding *Eo*-1-65, *Eo*-9 and *Eo*-4-5, the remaining 18 markers were used for population genetic studies. Genotypic linkage disequilibrium was not detected for any pair of markers in any populations.

**Table 1:** The genetic diversity estimated over 18 markers for populations of *E*. (*M*.) *onukii*.

Population	N	N_a_	N_e_	AR	H_O_	H_E_	Fis	F_null_
XY	30	9.3	5.35	7.470	0.704	0.767	0.083	0.043
RZ	30	9.0	5.00	7.200	0.737	0.760	0.030	0.030
TA	30	8.9	4.83	7.130	0.664	0.738	0.102	0.045
SX	30	9.7	5.93	7.860	0.699	0.780	0.107	0.062
CT	30	10.1	5.67	7.900	0.697	0.770	0.094	0.043
CX	30	9.3	4.96	7.390	0.700	0.754	0.074	0.042
PE	30	8.4	4.47	6.610	0.646	0.699	0.078	0.034
ZY	30	9.4	5.48	7.500	0.615	0.738	0.169	0.075
CY	30	10.4	5.89	8.270	0.668	0.788	0.155	0.065
JH	30	9.9	5.59	7.690	0.670	0.745	0.102	0.047
HZ	30	9.3	4.91	7.190	0.681	0.737	0.078	0.041
HS	30	9.7	5.19	7.580	0.665	0.733	0.095	0.039
NC	30	9.7	5.46	7.710	0.700	0.749	0.066	0.030
YT	30	8.4	5.00	6.960	0.713	0.745	0.044	0.031
CD	30	9.7	5.47	7.580	0.711	0.740	0.041	0.037
YD	30	9.7	5.49	7.440	0.656	0.733	0.106	0.050
GL	30	9.5	5.14	7.400	0.680	0.740	0.083	0.043
BS	30	8.8	4.89	6.960	0.635	0.702	0.098	0.041
FZ	30	9.8	5.65	7.680	0.707	0.745	0.052	0.027
JJ	30	8.4	4.19	6.520	0.632	0.665	0.051	0.039

N sample size, N_a_ mean number of alleles per marker, N_e_ mean effective number of alleles per marker, AR allelic richness, H_O_ observed heterozygosity, H_E_ expected heterozygosity, Fis inbreeding coefficient, F_null_ mean frequency of null allele per marker.

The number of alleles for the 18 microsatellites ranged from 2 to 18 over the entire population. Fifteen markers (all except *Eo*-42, *Eo*-1-57 and *Eo*-E-12) were highly polymorphic, with polymorphic information content (PIC) values ranging from 0.515 to 0.892^39^. At the population level, the mean N_a_ and AR ranged from 8.4 (PE, YT and JJ) to 10.4 (CY) and from 6.52 (JJ) to 8.27 (CY), respectively. The mean number of N_e_ per marker was 5.2, with the lowest value being 4.2 (JJ) and the highest being 5.9 (SX and CY). Only 69 individuals had one or more private alleles across all markers and they were spread across 17 populations. H_O_ had a mean value of 0.679 and was lowest in the ZY population (0.615) and highest in the RZ population (0.737). The mean H_E_ was 0.741, ranging from 0.665 (JJ) to 0.788 (CY) (Table 1). Although there is no significant difference in global mean AR and mean H_E_ among the four tea production areas (all P > 0.05), the mean AR was significantly different between the PE and the other populations in China (t-test: t = 2.395, d.f. = 17. P = 0.028). Similarly, the mean H_E_ was significantly different (t-test: t = 2.55, d.f. = 17. P = 0.021). The genetic diversity was significantly different between the CY population and the other populations (AR: t-test: t = −2.480, d.f. = 17. P = 0.024; H_E_: t-test: t = −2.172, d.f. = 17. P = 0.044). Overall genetic diversity was explained by 19 populations from China and one population from Japan also differing significantly in AR and H_E_ (China/Japan; AR: 7.419/6.520, P = 0.031; H_E_: 0.737/0.665, P = 0.002).

### Genetic structure

**Figure 1:**
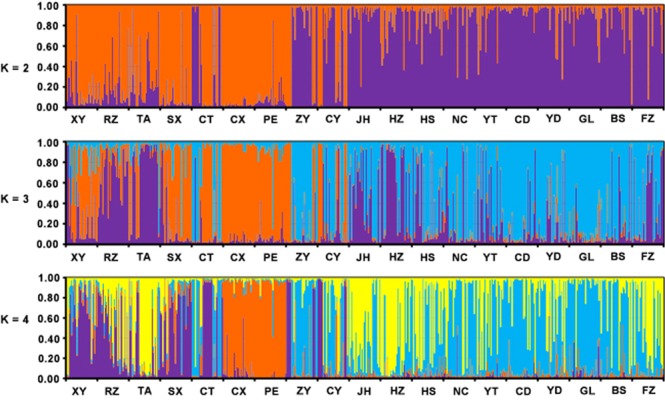
Structure of *E*. (*M*.) *onukii* populations in China revealed by Bayesian analysis implemented in Structure. Each individual is represented by a vertical bar broken into different colored genetic clusters, with length proportional to probability of assignment to each cluster. Analysis of 19 populations, 570 individuals, with possible numbers of clusters ranging from 2–4, indicated that the most likely number of clusters was 4.

The Bayesian analysis of population structure indicated that 19 populations from China represent four main genetic clusters (K = 4) (Fig. 1). Although ΔK had peaks in K = 2 and K = 4, α was relatively constant at K = 4. Furthermore, individuals were strongly assigned to K = 4. The CX population and PE population remain as a fixed cluster. The numbers of individuals from the CT population assigned to clusters are similar to those of the ZY and CY populations. The XY, RZ, TA and SX populations (all belonging to the Jiangbei tea production area) were assigned to each cluster similarly. The populations from Jiangnan and South China did not show strong population genetic structure.

Analysis of molecular variance (AMOVA) indicated that 2.43% of the genetic variation was partitioned among groups (*P* < 0.001) and 2.14% among populations (*P* < 0.001) (Table 2). The majority of the genetic variance originated from variation among individuals within populations (*P* < 0.001). The *F*_ST_ ranged from 0.0012 to 0.0981 among the populations. *F*_ST_ values between the JJ population and the other populations ranged from 0.0267 to 0.0981, revealing moderate differentiation. The largest range of *F*_ST_ was found in Southwest group 2, ranging from 0.0286 to 0.0981 (Supplementary Table S1). The mean *F*_ST_ values show moderate differentiation between Southwest group 2 and the other groups.

**Table 2:** AMOVA result of 20 *E*. (*M*.) *onukii* populations among five groups.

Source of variation	d.f.	Sum of squares	Variance components	Percentage of variation (%)	Fixation indices (P < 0.001)
Among groups	4	198.491	0.17019 Va	2.43	FCT = 0.024
Among populations within groups	15	233.398	0.14941 Vb	2.18	FSC = 0.022
Within population	1180	7791.967	6.67339 Vc	95.43	FST = 0.046
Total	1199	8223.856	6.993		

**Figure 2:**
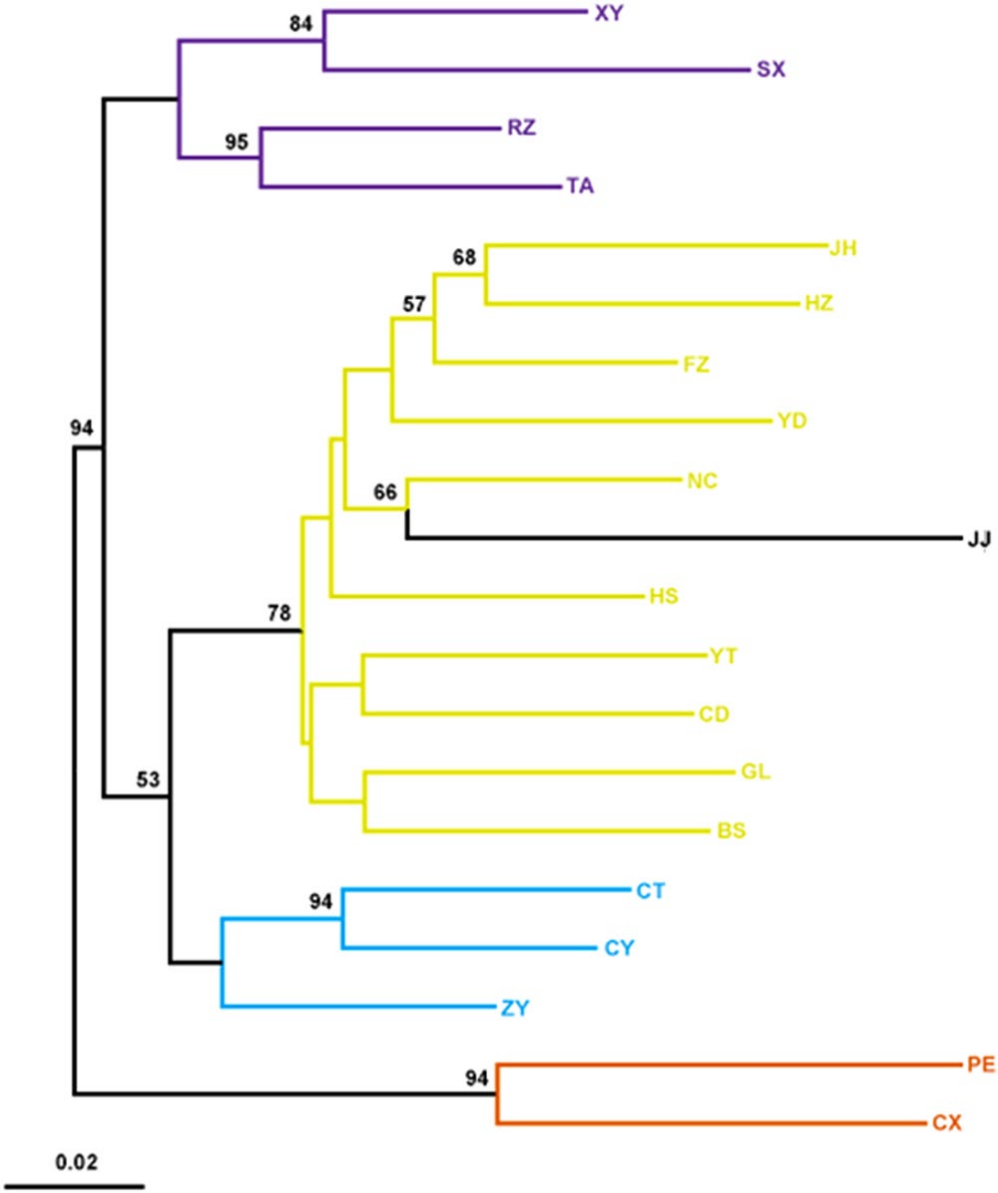
Neighbor-joining tree based on Nei’s genetic distances for 20 populations of *E*. (*M*.) *onukii* with allelic frequencies obtained from 18 microsatellite markers. Numbers on nodes represent bootstrap support values (values below 50% not shown). The colors indicate the major clusters inferred by Structure analysis when K = 4.

The NJT (Fig. 2) based on Nei’s genetic distance clustered the Chinese populations into 4 major groups, consistent with the results of both the Bayesian analysis and AMOVA. The populations from the Southeast tea production area were divided into two groups. The populations from the Jiangnan and South China tea production areas were clustered into one group. Nei’s genetic distances between Chinese populations and the Kagoshima population were 0.119–0.255 (Supplementary Table S2).

**Figure 3:**
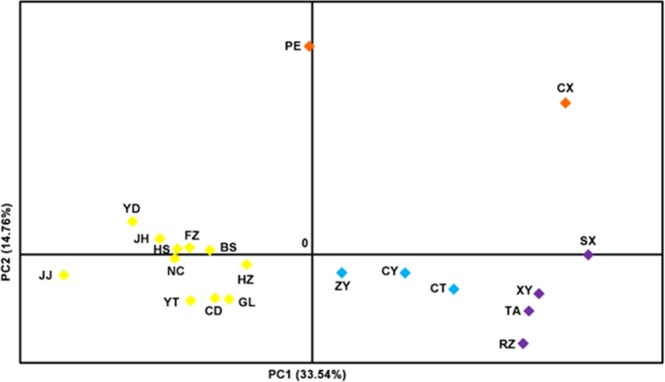
PCoA at population level generated from 18 microsatellite markers in 20 populations in China and Japan. The first two principle component factors, PC1 and PC2, account for 33.54% and 14.76% of total variance. Colors are coded as in Fig. 2.

Similar results were obtained in PCoA for these populations, as shown in Fig. 3. The mean factor scores for the 20 populations are shown in the first two principal component axes, which account for 33.54% and 14.76% of the total variance. The CX population is clearly distant from the PE population. This analysis shows conspicuous divergence of populations in the Jiangnan and South China tea production areas from the other populations.

Analyses of microsatellite data consistently indicate that Chinese *E*. (*M*.) *onukii* populations have differentiated into four groups: the Jiangbei group (purple populations), the Southwest group 1 (blue populations), the Southwest group 2 (orange populations) and the South China group (yellow populations) (Fig. 2).

### Isolation by distance

Mantel tests of genetic and geographical distances over all populations revealed that there was a significant correlation between these two variables (*R*_*XY*_ = 0.549, *R*2 = 0.301, *P = *0.000, Supplementary Fig. S1a). However, it was not significant when only the Jiangbei populations (*R*_*XY*_ = 0.495, *R*^2^ = 0.245, *P* = 0.318) and Southwest populations (*R*_*XY*_ = 0.609, *R*^2^ = 0.371, *P* = 0.062) were analyzed. There was a significant correlation when the South China group (including Jiangnan and South China populations) (*R*_*XY*_ = 0.539, *R*^2^ = 0.291, *P* = 0.0001, Supplementary Fig. S1b) was analyzed.

### Bottleneck analysis

Significant heterozygosity excess was detected in 4 of 20 populations (HS, YT, GL, FZ) under the SMM model. The TPM model revealed significant evidence of heterozygosity excess (P < 0.05) in XY, South China and most of the Jiangnan populations except for the CD (P = 0.054) and JH (P = 0.142) populations, which may indicate a recent genetic bottleneck^40^ in these populations. In contrast, most of the Jiangbei, Southwest and Kagoshima populations show significant heterozygosity deficiency, indicating that these populations have been expanding^40^.

## Discussion

Our analysis of microsatellite data reveals that genetic differentiation of *E*. (*M*.) *onukii* within a particular region is mainly reflected in the differences among individuals within a population. This is consistent with previous results from AMOVA based on mtDNA *COI* and 16S rRNA^21–25^. Analysis of microsatellite markers detected low to moderate levels of genetic differentiation among populations within the main tea growing regions of China, and much more genetic variation within populations (95.43%) than among populations (2.18%). This suggests substantial amounts of gene flow and a more homogeneous gene pool across different geographical populations. Measured genetic and geographical distances are correlated. Within a population, genetic mutation and drift are expected to yield genetic differentiation among individuals, whereas geographic isolation (such as geographic distance and geographic barriers) mainly influences genetic differentiation between populations.

The low to moderate levels of genetic differentiation and subdivision are concordant with geographic distribution. The genetic structure based on 18 microsatellite markers confirms the genetic difference between Southwest groups 1 and 2 in the topographically complex Southwest tea production area, similar to the result obtained previously^27^. Furthermore, different allelic richness and mean expected heterozygosity were observed in the PE and CY populations of the Southwest tea production area (Tables 1 and 2). There was moderate differentiation between populations of Southwest group 2 and the other populations. Genetic diversity differs significantly between the CY and CT populations and other populations, which are isolated by the Sichuan Basin (AR: t-test: t = −2.944, d.f. = 17. P = 0.009; H_E_: t-test: t = −2.582, d.f. = 17. P = 0.019). These subdivisions in the Southwest tea production area populations are probably due to different combinations of geographic isolation and climatic variation. Substantial climatic differences between the Southwest tea production area and the other tea production areas and the existence of geographic barriers in this area, such as mountain ranges, basins and large rivers, may account for the genetic differentiation among *E*. (*M*.) *onukii* populations that have limited dispersal capacity^22,23^.

Fossil evidence suggests that tea trees originated 60–70 Mya (million years ago) in the Paleogene of the Yunnan-Guizhou plateau^41^. Terpene index analysis has shown that the original tea cultivars are from Yunnan Province^42^. This Southwest tea production area was previously suggested as the area in which tea was first cultivated. The “original” haplotypes of 16S rRNA of *E*. (*M*.) *onukii* (based on a median joining haplotype network) were obtained from Yunnan and Sichuan populations^22^, suggesting that the tea green leafhopper expanded its range from the Southwest tea production area into other areas, following the spread of tea cultivation. Based on this evidence, as well as the genetic diversity and structure inferred in this study, we propose the following scenario for the spread of *E*. (*M*.) *onukii* populations in Chinese tea production areas: (1) the tea green leafhopper spread from Yunnan to Guangxi and Guangdong, and subsequently to Jiangnan and the South China tea production areas; (2) this pest then expanded to the Jiangbei tea production area from Yunnan through Shaanxi; and (3) the leafhopper was introduced to Jiangnan and the South China tea production areas from Yunnan through Guizhou, Chongqing and Sichuan provinces. The genetic pattern of *E*. (*M*.) *onukii* is similar to the historical propagation path of tea in China^42^. Low genetic diversity and moderate genetic differentiation of *E*. (*M*.) *onukii* observed in the Kagoshima population of Japan may have resulted from a bottleneck caused by anthropogenic transport of tea plants, geographical isolation and restricted genetic resources^43,44^. Based on Nei’s genetic identity >0.85 the Kagoshima population is closest to the populations in Jiangnan and South China. Similar tea germplasms in these populations also provide similar biotopes for this pest^45^.

This study suggests that different populations of *E*. (*M*.) *onukii* are currently undergoing population expansion in the South China and Jiangnan tea production areas but are experiencing bottlenecks in other tea production areas. Mating interference and trapping of males may be used to keep *E*. (*M*.) *onukii* from spreading into surrounding tea production areas beyond Kagoshima, Southwest China and most of the Jiangbei populations. The South China and most of the Jiangnan populations, may show the effect of a recent genetic bottleneck, possibly resulting from heavy use of chemical pesticides. In these regions, only a few resistant individuals may have survived, leading to the genetic bottleneck effects, similar to those previously reported for *Bemisia tabaci* and *Myzus persicae*^46,47^. In these cases, pesticides may have stimulated the reproduction and expansion of pest populations, promoting gene exchange and homogenizing the genetic structure^48^. Integrative pest management incorporating biocontrol or organic production methods may be needed to build ecologically sustainable tea plantations in these areas^49,50^.

Microsatellite markers revealed lower genetic differentiation between Sichuan and Yunnan populations than was shown in a previous study on this same species in the same region^27^. This may be because the present study used samples obtained from multiple localities in Sichuan. To provide more robust results, future studies should employ larger numbers of genetic markers and more individuals within the same locations to more fully represent the diversity of local *E*. (*M*.) *onukii* populations. The area of origin of *E*. (*M*.) *onukii* should be further explored by more intensive sampling of populations in the Southwest tea production area, where the level of genetic differentiation is highest. The dispersal routes of *E*. (*M*.) *onukii* among Chinese tea production areas should also be analyzed in more detail using more genetic loci (such as SNPs). Use of SNPs that have a high abundance throughout the genome, predictable mutation modes and low back mutation rates^51,52^ could reduce error in estimation of population parameters (such as *F*_ST_ and migration rates) compared to our use of microsatellite markers.

This study found low to moderate levels of genetic differentiation and segregation in *E*. (*M*.) *onukii* populations among the main Chinese tea production areas. Populations of this pest have differentiated into four groups: the Jiangbei group, the Southwest group 1, the Southwest group 2 and the South China group. Low genetic diversity and moderate genetic differentiation of *E*. (*M*.) *onukii* were found in the Kagoshima population of Japan. The observed patterns may be attributable to geographic isolation, differences in feeding preference among tea germplasms and the use of pesticides in tea production areas. Additional study is needed to elucidate the relationship between selective factors (such as tea germplasm resources and geographical conditions) and genetic structure.

## Materials and Methods

### Sampling

**Figure 4:**
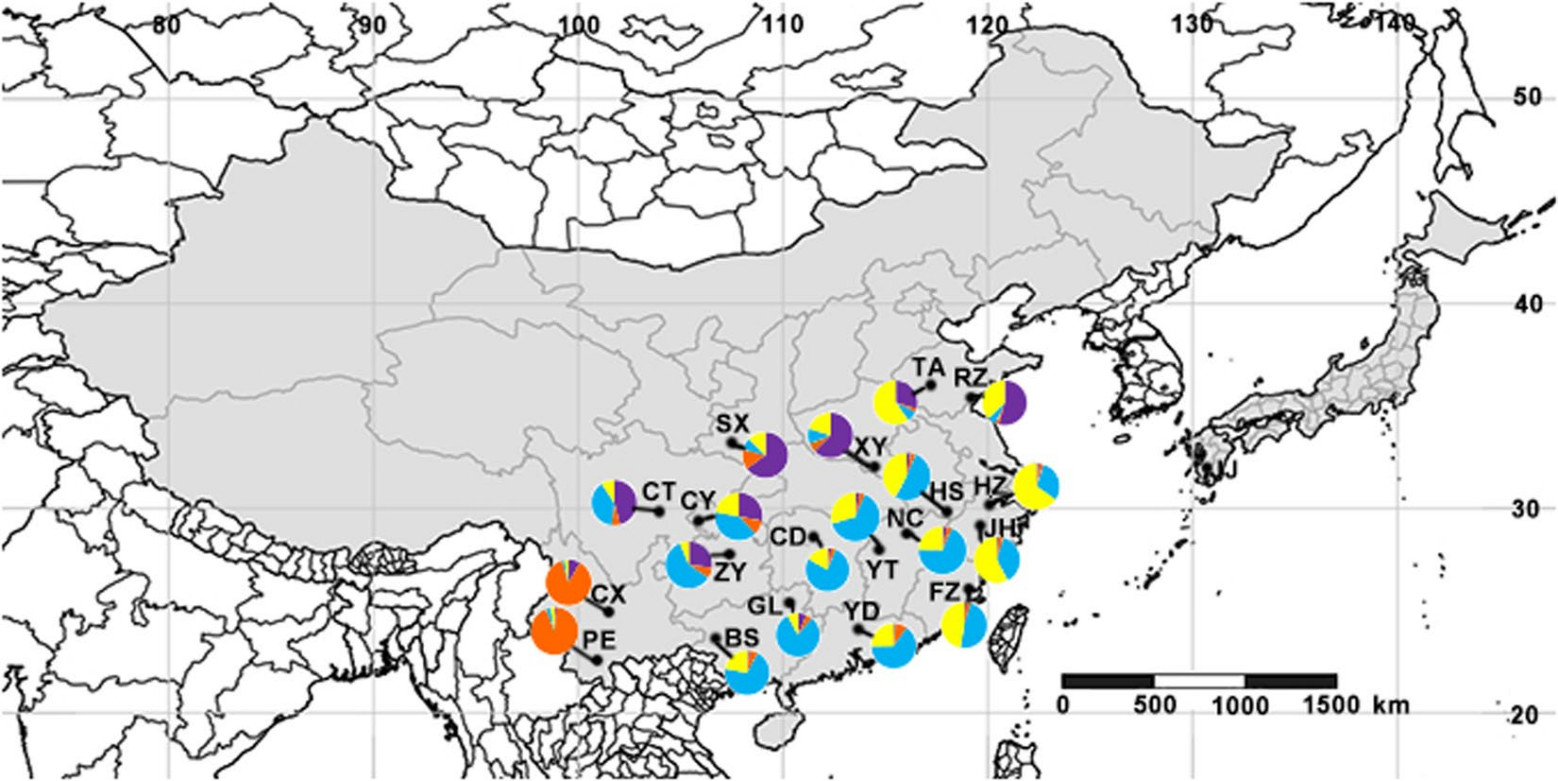
Geographic distribution and Bayesian model-based cluster analysis of *E*. (*M*.) *onukii*. Population codes are listed in Table 3; the pie chart in each population represents the proportion of individuals from four clusters inferred by Structure analysis. SimpleMappr was used to produce a distribution map based on the geographical coordinates in Table 3. URL: http://www.simplemappr.net/#tabs=0.

*E*. (*M*.) *onukii* were collected by sweep net from 19 localities in four tea production areas of China and one location in Japan, representing a total of 20 geographically delimited populations (Fig. 4 and Table 3). In order to reduce the likelihood of sample contamination from non-tea plants, specimens obtained in each tea garden were collected from five different sites (approximately five square meters per site) selected at random near the middle of each plantation. The net was swept across the top of the tea plants at each site and the collected specimens were placed in five separate vials. All specimens were preserved in absolute alcohol at −20 °C until they were identified and used for genotyping. The rest of the specimens are now deposited in the Entomological Museum, Northwest A&F University, Yangling, China (NWAFU).

**Table 3:** Description of the populations collected in China and Japan.

Population No. & code	Collecting locality		Tea area	Latitude(N)/Longitude(E)	Collection date (M/Y)
1 XY	Henan	Xinyang	Jiangbei	32.09°/114.06°	7/2013
2 RZ	Shandong	Rizhao	Jiangbei	35.29°/119.26°	7/2013
3 TA		Taian	Jiangbei	36.17°/117.24°	8/2013
4 SX	Shaanxi	Hanzhong	Jiangbei	32.98°/107.77°	6/2016
5 CT	Sichuan	Leshan	Southwest	29.79°/103.69°	8/2013
6 CX	Yunnan	Chuxiong	Southwest	24.57°/101.81°	7/2014
7 PE		Puer	Southwest	22.75°/100.96°	7/2015
8 ZY	Guizhou	Zunyi	Southwest	27.77°/107.48°	7/2014
9 CY	Chongqing	Yongchuan	Southwest	29.40°/105.92°	5/2014
10 JH	Zhejiang	Jinhua	Jiangnan	28.89°/119.82°	9/2014
11 HZ		Hangzhou	Jiangnan	30.21°/120.09°	9/2014
12 HS	Anhui	Huangshan	Jiangnan	29.85°/117.72°	9/2014
13 NC	Jiangxi	Nanchang	Jiangnan	28.81°/115.72°	7/2014
14 YT		Yichun	Jiangnan	28.52°/114.37°	7/2014
15 CD	Hunan	Changde	Jiangnan	28.64°/111.16°	7/2014
16 YD	Guangdong	Yingde	South China	24.30°/113.40°	7/2015
17 GL	Guangxi	Guilin	South China	25.28°/110.34°	7/2015
18 BS		Baise	South China	24.50°/106.66°	7/2015
19 FZ		Fuzhou	South China	26.08°/119.24°	5/2014
20 JJ	Japan	Kagoshima	—	31.60°/130.56°	8–10/2014

Only male specimens representing each population were selected for this study because of the inability to identify females using morphological characters^1^. All were identified in advance in the laboratory by the first author based on morphological characters diagnostic for the species^1^.

### DNA extraction and microsatellite genotyping

Total genomic DNA was extracted respectively from 30 male individuals per population (except for genital segments used for species identification) using the DNA Easy Blood and Tissue Kit (Qiagen, Hilden, Germany) or a modified CTAB method^53^. DNA concentration was measured using an ND-1000 spectrophotometer (Bio-Rad, Hercules, CA, USA). Eighteen microsatellite markers (Supplementary Table S3) out of the 21 originally characterized by Zhang *et al*.^27^ were labeled with a fluorescent dye (HEX, TAMRA or FAM). PCR protocol was performed as described by Zhang *et al*.^27^. PCR products were run by automated capillary electrophoresis on an ABI 3130xl (Applied Biosystems, Foster, CA, USA) genetic analyzer. Allele sizes were scored against a GeneScan^TM^ 500LIZ standard using GeneMapper v4.0 (Applied Biosystems, Foster, CA, USA).

### Genetic diversity analysis

FreeNA was used to evaluate the frequency of null alleles (F_null_)^37^. Allele frequency, observed and expected heterozygosity were calculated with Genepop v.4.2 for each marker by the allele sizes from each geographical population. Deviations from Hardy-Weinberg equilibrium (HWE) were tested with 1000 permutations across markers using Genepop v.4.2^54^. Linkage disequilibrium (LD) for each pair of markers in the populations was assessed with Genepop v.4.2. Statistical significance (P) was corrected for multiple comparisons by applying the strict Bonferroni correction^55^ with the same software.

Mean observed (H_O_) and expected heterozygosity (H_E_), mean effective number of alleles per marker (N_e_), mean number of alleles (N_a_) per marker and polymorphism information content (PIC) were calculated using GenALEx v. 6.5^56^ and Cervus 2.0^57^. The allelic richness (AR) was estimated using a minimum sample size of 26 diploid individuals in HP-Rare v1.0^58^. Statistical significance of the genetic diversity indices between different populations was assessed using the independent samples *t*-test in SPSS Statistics 20 (IBM).

### Genetic structure analysis

Bayesian analysis, Analysis of Molecular Variance (AMOVA), phylogenetic analysis using the Neighbor Joining (NJ) approach and Principal Coordinate Analysis (PCoA) were used to analyze the genetic structure of the tea green leafhopper. First, Bayesian model-based clustering analysis implemented in Structure v2.3.4 was used to estimate the number of genetically distinct clusters (K) among the Chinese populations using an admixture ancestry model and correlated allele frequencies. The range of possible clusters was set from 1 to 10, with 20 independent runs for each K. Analysis parameters included a burn-in period of 50,000 iterations followed by 1,000,000 Markov Chain Monte Carlo (MCMC) repetitions. The most likely number of genetic clusters (K) in the Chinese populations was estimated by posterior probability of the data (P (K/X)), the Dirichlet parameter Alpha (α) and the ad hoc statistic (ΔK)^59–62^. The output from Structure was visualized in DISTRUCT version 1.1^63^. Second, an analysis of molecular variance (AMOVA) was performed using Arlequin 3.11 to estimate and compare the percentage of genetic variation explained by different hierarchical levels: individual (within populations, i.e., among leafhoppers in the same population), populations (i.e., among populations in the same tea production areas) and groups (i.e. Jiangbei group included XY, RZ, TA and SX populations; Southwest group 1 included CT, ZY and CY populations; Southwest group 2 included CX and PE populations; South China group included JH, HZ, HS, NC, YT, CD, YD, GL, BS and FZ populations; Japan group included JJ population). Arlequin 3.11 with 1000 permutations was also used to calculate pairwise estimates of *F*_ST_ and the significance (*P*) for each population^64^. The levels of differentiation between populations were measured based on *F*_ST_^65^. Third, the genetic distances between populations were measured by *D*_*A*_ distance in POPTREE2^66,67^. A neighbor-joining tree (NJT) was constructed with POPTREE2 based on *D*_*A*_ distance and 1000 replications. Finally, a principal coordinate analysis (PCoA) was carried out using GenALEX 6.502 based on Phi-st distances (GD) of populations^56^.

### Isolation by distance

Isolation by distance was analyzed in GenALEX 6.502 to detect the relationship between genetic and geographic distances^56^. Geographic distance was defined as the logarithms of the geographical distance between all pairs of population locations, and genetic distance was defined by pairwise population estimates of linearized *F*_ST_/(1 − *F*_ST_). The correlation between the two parameter matrices was regressed using a Mantel test, with the significance estimated by *P* value, and the regression coefficient (*R*) and the mean correlation coefficient (*R*_XY_) performed over 999 random permutations.

### Bottleneck analysis

Bottleneck version 1.2.02^40^ was used to test whether the populations experienced a recent bottleneck or expansion. The observed heterozygosity was compared with the expected heterozygosity under a two-phase mutation model (TPM) recommended for microsatellite data^68^ and the step-wise-mutation model (SMM) (TPM = 95% SMM; a variance among multiple steps of 12). The significance of excess heterozygosity was assessed using a Wilcoxon test (5000 iterations).

## Supplementary information

Supplementary Fig. S1; Supplementary Table S1; Supplementary Table S2; Supplementary Table S3

## Data Availability

All data generated or analysed during current study are available within the published article (and its Supplementary Information files).
