# Supplementary material for: Population structure and genetic differentiation of tea green leafhopper, Empoasca (Matsumurasca) onukii, in China based on microsatellite markers
Source: Sci Rep. 2019 Feb 4;9:1202. doi: 10.1038/s41598-018-37881-0 (PMC6361905; doi:10.1038/s41598-018-37881-0)
Supplement: Supplementary file 1 — Supplementary Fig. S1; Supplementary Table S1; Supplementary Table S2; Supplementary Table S3 [file 41598_2018_37881_MOESM1_ESM.doc]

**Supplementary information**

**Population structure and genetic differentiation** **of tea green leafhopper, *Empoasca* (*Matsumurasca*) *onukii*, in China based on microsatellite markers**

Li Zhang^1^, Fuping Wang^2^, Li Qiao^3^, Christopher H. Dietrich^4^, Masaya Matsumura^5^, Daozheng Qin^1^*

^1^ Key Laboratory of Plant Protection Resources and Pest Management of the Ministry of Education; Entomological Museum, Northwest A&F University, Yangling, Shaanxi 712100, China

^2^ Yangling Xianglin Agricultural Science & Technology Chemical Company Limited, Yangling, Shaanxi 712100, China

^3^ College of Agronomy, Xinyang Agricultural and Forestry University, Xinyang, Henan 464000, China

^4^ Illinois Natural History Survey, Prairie Research Institute, University of Illinois, 1816 S. Oak Street, Champaign, IL 61820, U.S.A.

^5^ Department of Planning and Coordination, Headquarters, National Agriculture and Food Research Organization, 3-1-1 Kannondai, Tsukuba, Ibaraki, 305-8517, Japan.

*Correspondence

***Corresponding Author:**

Dr. Dao-Zheng Qin

Email: [qindaozh0426@aliyun.com](mailto:qindaozh0426@aliyun.com)

Tel: 86-29-87092524

Postal Address: Key Laboratory of Plant Protection Resources and Pest Management of the Ministry of Education; Entomological Museum, Northwest A&F University, Yangling, Shaanxi 712100, China

**Figure Legends**

**Supplementary Fig. S1** Correlation analysis between pairwise population estimates of linearized *F*_ST_/(1-*F*_ST_) and the logarithms of geographic distance in Chinese populations of *E*. (*M*.) *onukii* based on microsatellites (a) total populations in China; (b) South China group (including Jiangnan and South China populations).

**Supplementary Fig. S1**

**
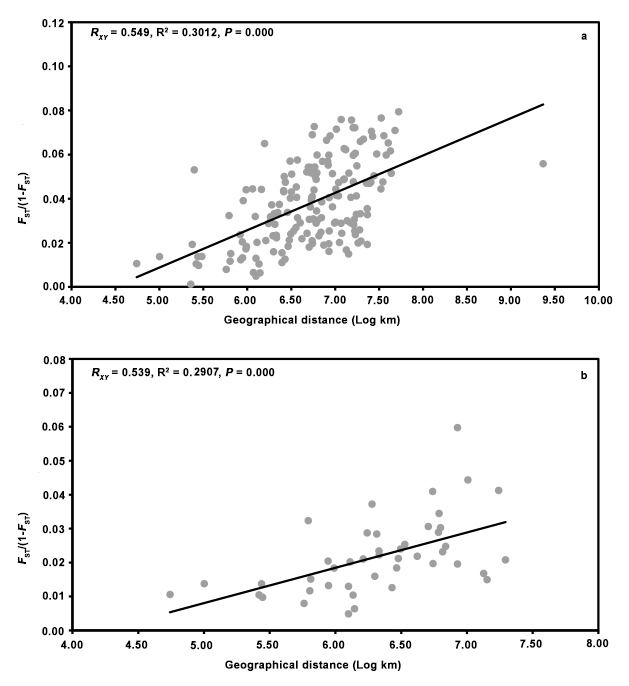
**

**Supplementary Table S1** Pairwise estimates of *F*_ST_ (below diagonal) and the associated *P*-values

|  | XY | RZ | TA | SX | CT | CX | PE | ZY | CY | JH | HZ | HS | NC | YT | CD | YD | GL | BS | FZ | JJ |
| --- | --- | --- | --- | --- | --- | --- | --- | --- | --- | --- | --- | --- | --- | --- | --- | --- | --- | --- | --- | --- |
| XY |  | ** | ** | ** | ** | ** | ** | ** | ** | ** | ** | ** | ** | ** | ** | ** | ** | ** | ** | ** |
| RZ | 0.0153 |  | ** | ** | ** | ** | ** | ** | ** | ** | ** | ** | ** | ** | ** | ** | ** | ** | ** | ** |
| TA | 0.0227 | 0.0189 |  | ** | ** | ** | ** | ** | ** | ** | ** | ** | ** | ** | ** | ** | ** | ** | ** | ** |
| SX | 0.0109 | 0.0282 | 0.0304 |  | ** | ** | ** | ** | ** | ** | ** | ** | ** | ** | ** | ** | ** | ** | ** | ** |
| CT | 0.0159 | 0.0189 | 0.0252 | 0.0290 |  | ** | ** | NS | ** | ** | ** | ** | ** | ** | ** | ** | ** | ** | ** | ** |
| CX | 0.0321 | 0.0581 | 0.0564 | 0.0286 | 0.0418 |  | ** | ** | ** | ** | ** | ** | ** | ** | ** | ** | ** | ** | ** | ** |
| PE | 0.0659 | 0.0736 | 0.0663 | 0.0703 | 0.0514 | 0.0504 |  | ** | ** | ** | ** | ** | ** | ** | ** | ** | ** | ** | ** | ** |
| ZY | 0.0237 | 0.0319 | 0.0390 | 0.0361 | 0.0065 | 0.0487 | 0.0421 |  | ** | ** | ** | ** | ** | ** | ** | ** | ** | ** | ** | ** |
| CY | 0.0205 | 0.0195 | 0.0282 | 0.0310 | 0.0012 | 0.0413 | 0.0465 | 0.0136 |  | ** | ** | ** | ** | ** | ** | ** | ** | ** | ** | ** |
| JH | 0.0540 | 0.0544 | 0.0646 | 0.0586 | 0.0449 | 0.0712 | 0.0613 | 0.0466 | 0.0296 |  | ** | ** | ** | ** | ** | ** | ** | ** | ** | ** |
| HZ | 0.0414 | 0.0326 | 0.0304 | 0.0464 | 0.0343 | 0.0642 | 0.0490 | 0.0292 | 0.0232 | 0.0136 |  | ** | ** | ** | ** | ** | ** | ** | ** | ** |
| HS | 0.0426 | 0.0453 | 0.0434 | 0.0539 | 0.0275 | 0.0639 | 0.0425 | 0.0248 | 0.0198 | 0.0136 | 0.0097 |  | ** | ** | ** | ** | ** | ** | NS | ** |
| NC | 0.0422 | 0.0495 | 0.0492 | 0.0492 | 0.0281 | 0.0619 | 0.0449 | 0.0177 | 0.0186 | 0.0180 | 0.0198 | 0.0104 |  | ** | ** | ** | ** | ** | ** | ** |
| YT | 0.0376 | 0.0494 | 0.0477 | 0.0515 | 0.0351 | 0.0674 | 0.0628 | 0.0262 | 0.0299 | 0.0279 | 0.0218 | 0.0149 | 0.0105 |  | ** | ** | ** | ** | ** | ** |
| CD | 0.0423 | 0.0489 | 0.0524 | 0.0529 | 0.0283 | 0.0641 | 0.0590 | 0.0122 | 0.0309 | 0.0393 | 0.0281 | 0.0208 | 0.0128 | 0.0115 |  | ** | ** | ** | ** | ** |
| YD | 0.0516 | 0.0563 | 0.0673 | 0.0668 | 0.0452 | 0.0706 | 0.0491 | 0.0390 | 0.0371 | 0.0297 | 0.0242 | 0.0214 | 0.0276 | 0.0206 | 0.0359 |  | ** | ** | ** | ** |
| GL | 0.0390 | 0.0521 | 0.0572 | 0.0565 | 0.0350 | 0.0678 | 0.0623 | 0.0170 | 0.0327 | 0.0564 | 0.0425 | 0.0333 | 0.0234 | 0.0229 | 0.0131 | 0.0313 |  | ** | ** | ** |
| BS | 0.0399 | 0.0480 | 0.0454 | 0.0539 | 0.0387 | 0.0610 | 0.0477 | 0.0231 | 0.0322 | 0.0396 | 0.0204 | 0.0165 | 0.0192 | 0.0227 | 0.0181 | 0.0247 | 0.0200 |  | ** | ** |
| FZ | 0.0379 | 0.0389 | 0.0397 | 0.0455 | 0.0317 | 0.0569 | 0.0454 | 0.0238 | 0.0248 | 0.0079 | 0.0063 | 0.0049 | 0.0103 | 0.0157 | 0.0193 | 0.0125 | 0.0294 | 0.0147 |  | ** |
| JJ | 0.0701 | 0.0788 | 0.0867 | 0.0876 | 0.0613 | 0.0981 | 0.0806 | 0.0477 | 0.0594 | 0.0475 | 0.0553 | 0.0370 | 0.0267 | 0.0370 | 0.0526 | 0.0401 | 0.0483 | 0.0385 | 0.0343 |  |

P-values are obtained with 1000 iterations. **represents the significant differences and NS means not significant.

**Supplementary Table S2** Pairwise estimates of *D_A_* (below diagonal) and pairwise estimates of genetic identity (above diagonal) between populations in China and Japan

|  | XY | RZ | TA | SX | CT | CX | PE | ZY | CY | JH | HZ | HS | NC | YT | CD | YD | GL | BS | FZ | JJ |
| --- | --- | --- | --- | --- | --- | --- | --- | --- | --- | --- | --- | --- | --- | --- | --- | --- | --- | --- | --- | --- |
| XY | **** | 0.922 | 0.907 | 0.936 | 0.919 | 0.873 | 0.785 | 0.902 | 0.899 | 0.802 | 0.849 | 0.846 | 0.840 | 0.856 | 0.843 | 0.816 | 0.853 | 0.868 | 0.852 | 0.804 |
| RZ | 0.095 | **** | 0.920 | 0.875 | 0.911 | 0.784 | 0.759 | 0.880 | 0.906 | 0.804 | 0.877 | 0.839 | 0.818 | 0.821 | 0.825 | 0.804 | 0.813 | 0.846 | 0.850 | 0.782 |
| TA | 0.109 | 0.078 | **** | 0.879 | 0.900 | 0.805 | 0.794 | 0.865 | 0.889 | 0.784 | 0.891 | 0.854 | 0.830 | 0.838 | 0.825 | 0.782 | 0.809 | 0.861 | 0.857 | 0.769 |
| SX | 0.099 | 0.138 | 0.140 | **** | 0.869 | 0.881 | 0.768 | 0.862 | 0.854 | 0.778 | 0.827 | 0.805 | 0.811 | 0.806 | 0.803 | 0.759 | 0.791 | 0.825 | 0.821 | 0.753 |
| CT | 0.122 | 0.117 | 0.128 | 0.139 | **** | 0.834 | 0.827 | 0.956 | 0.967 | 0.832 | 0.871 | 0.894 | 0.886 | 0.865 | 0.889 | 0.837 | 0.867 | 0.870 | 0.872 | 0.831 |
| CX | 0.165 | 0.192 | 0.192 | 0.156 | 0.186 | **** | 0.835 | 0.827 | 0.828 | 0.750 | 0.780 | 0.781 | 0.779 | 0.762 | 0.776 | 0.757 | 0.763 | 0.808 | 0.795 | 0.727 |
| PE | 0.223 | 0.236 | 0.219 | 0.218 | 0.203 | 0.128 | **** | 0.864 | 0.840 | 0.810 | 0.848 | 0.867 | 0.857 | 0.810 | 0.818 | 0.848 | 0.802 | 0.863 | 0.855 | 0.794 |
| ZY | 0.131 | 0.122 | 0.141 | 0.155 | 0.084 | 0.199 | 0.198 | **** | 0.934 | 0.840 | 0.895 | 0.908 | 0.925 | 0.899 | 0.943 | 0.866 | 0.929 | 0.919 | 0.907 | 0.871 |
| CY | 0.125 | 0.115 | 0.135 | 0.145 | 0.078 | 0.185 | 0.208 | 0.106 | **** | 0.879 | 0.905 | 0.917 | 0.913 | 0.875 | 0.877 | 0.859 | 0.871 | 0.889 | 0.892 | 0.834 |
| JH | 0.163 | 0.144 | 0.170 | 0.193 | 0.165 | 0.225 | 0.206 | 0.158 | 0.137 | **** | 0.938 | 0.939 | 0.923 | 0.891 | 0.861 | 0.892 | 0.808 | 0.873 | 0.953 | 0.871 |
| HZ | 0.161 | 0.118 | 0.131 | 0.179 | 0.156 | 0.229 | 0.216 | 0.134 | 0.135 | 0.094 | **** | 0.951 | 0.920 | 0.913 | 0.897 | 0.911 | 0.855 | 0.928 | 0.958 | 0.852 |
| HS | 0.141 | 0.129 | 0.119 | 0.169 | 0.128 | 0.217 | 0.206 | 0.117 | 0.110 | 0.103 | 0.097 | **** | 0.947 | 0.933 | 0.919 | 0.919 | 0.883 | 0.937 | 0.964 | 0.899 |
| NC | 0.158 | 0.142 | 0.146 | 0.172 | 0.143 | 0.222 | 0.199 | 0.116 | 0.135 | 0.111 | 0.121 | 0.095 | **** | 0.943 | 0.939 | 0.897 | 0.908 | 0.927 | 0.946 | 0.925 |
| YT | 0.149 | 0.146 | 0.153 | 0.173 | 0.147 | 0.221 | 0.220 | 0.118 | 0.153 | 0.130 | 0.120 | 0.112 | 0.101 | **** | 0.941 | 0.917 | 0.908 | 0.922 | 0.928 | 0.901 |
| CD | 0.172 | 0.145 | 0.154 | 0.186 | 0.123 | 0.227 | 0.219 | 0.093 | 0.146 | 0.142 | 0.127 | 0.108 | 0.105 | 0.097 | **** | 0.875 | 0.940 | 0.932 | 0.921 | 0.859 |
| YD | 0.174 | 0.167 | 0.191 | 0.200 | 0.164 | 0.231 | 0.205 | 0.149 | 0.147 | 0.111 | 0.123 | 0.118 | 0.118 | 0.119 | 0.130 | **** | 0.888 | 0.917 | 0.941 | 0.891 |
| GL | 0.161 | 0.161 | 0.169 | 0.193 | 0.142 | 0.224 | 0.223 | 0.110 | 0.138 | 0.158 | 0.152 | 0.118 | 0.121 | 0.118 | 0.108 | 0.119 | **** | 0.928 | 0.888 | 0.869 |
| BS | 0.165 | 0.153 | 0.147 | 0.198 | 0.163 | 0.222 | 0.201 | 0.127 | 0.139 | 0.137 | 0.105 | 0.109 | 0.111 | 0.127 | 0.107 | 0.115 | 0.103 | **** | 0.942 | 0.898 |
| FZ | 0.162 | 0.141 | 0.141 | 0.183 | 0.153 | 0.218 | 0.214 | 0.133 | 0.140 | 0.085 | 0.093 | 0.085 | 0.096 | 0.114 | 0.110 | 0.093 | 0.130 | 0.103 | **** | 0.905 |
| JJ | 0.196 | 0.172 | 0.188 | 0.228 | 0.185 | 0.255 | 0.244 | 0.153 | 0.186 | 0.159 | 0.169 | 0.138 | 0.119 | 0.147 | 0.161 | 0.141 | 0.153 | 0.151 | 0.128 | **** |

**Supplementary Table S3 Characteristics of 18 microsatellite markers analyzed in *E*. (*M*.) *onukii***

| Markers (GenBank accession no.) | Repeat | Primer sequence (5'–3') | Size range (bp) | Observed number of alleles |
| --- | --- | --- | --- | --- |
| *Eo*-29 | (TC)_6_ | F:[TAMRA]-CCAGTGAGGAAAGGAGGA | 140-176 | 16 |
| (KU588268) |  | R:GGGTATGATTAGCGGTGT |  |  |
| *Eo*-51 | (CT)_11_GT(CT)_4_ | F:[FAM]-TCGCTCCACTCTACCACT | 188-246 | 28 |
| (KU588269) |  | R:CTAAAACTAACAAATCCACCT |  |  |
| *Eo*-37 | (AG)_6_ | F:[TAMRA]-GTTTTGGGTATGATTAG | 153-181 | 14 |
| (KU588270) |  | R:CCAGTGAGGAAAGGAG |  |  |
| *Eo*-54 | (GA)_8_ | F:[FAM]-CTGTTCGCAGTTCACATCATTC | 399-459 | 24 |
| (KU588271) |  | R:GACCCGCTACGCTTACCTATT |  |  |
| *Eo*-1-61 | (CA)_4_…(CA)_4_ | F:[HEX]-CGGCATTCATTATCTC | 82-112 | 16 |
| (KU588272) |  | R:CACACAACTCACTCGCT |  |  |
| *Eo*-1-52 | (AC)_12_ | F:[FAM]-GCCGTGTGTAATGGTATCC | 195-247 | 22 |
| (KU588273) |  | R:GACGCCTAGCAATGTT |  |  |
| *Eo*-42 | (GA)_8_ | F:[HEX]-GGACAATGAAAATTCGAGGACGG | 111-179 | 29 |
| (KU588274) |  | R:GGCAATCGGCAACAACAAAC |  |  |
| *Eo*-1-82 | (GT)_14_ | F:[FAM]-TGACAGCCATAAACACCG | 264-308 | 23 |
| (KU588275) |  | R:CGTAGACCAGATGACCCTC |  |  |
| *Eo*-20 | (GA)_9_ | F:[TAMRA]-CTGTCACTTGCCAATAACTCT | 127-169 | 19 |
| (KU588277) |  | R:CAACCACCTCACTCCCTCT |  |  |
| *Eo*-1-57 | (AC)_6_ | F:[HEX]-TACTCACCGCTCGTCTATC | 93-111 | 8 |
| (KU588279) |  | R:CACTTTTATTTTCGGCTCT |  |  |
| *Eo*-83 | (AG)_6_ | F:[HEX]-GAGTCTGTTCGGTTTGATGT | 90-132 | 12 |
| (KU588280) |  | R:CAAGCGATAAGCAAGGTAAG |  |  |
| *Eo*-E-12 | (GT)_14_ | F:[TAMRA]-GCAAGCCGTTAGCATAGT | 123-185 | 27 |
| (KU588281) |  | R:AACACCGCATTTCATACA |  |  |
| *Eo*-70 | (AC)_9_C(CA)_12_ | F:[FAM]-CAGGAGCAGGACAAGAG | 179-269 | 29 |
| (KU588282) |  | R:GCACATAAGCCTAAACAGAC |  |  |
| *Eo*-36 | (AG)_9_ | F:[FAM]-CCAGACAGCGAAGTGAAT | 257-323 | 22 |
| (KU588284) |  | R:GGTACCCGAAGGAAGGAT |  |  |
| *Eo*-68 | (AG)_4_GG(AG)_3_ | F:[TAMRA]-TAGGGGTTCGACAGACTTG | 146-198 | 23 |
| (KU588285) |  | R:GAGGTGAGGTTGGGATTTG |  |  |
| *Eo*-1-5 | (GTT)_4_ | F:[TAMRA]-CCGAAGAAGTCCAAGATAA | 121-148 | 8 |
| (KU588288) |  | R:CGAGGAGGAGGCTAAAG |  |  |
| *Eo*-F-8 | (CA)_13_G(AC)_5_ | F:[HEX]-CCTTGTAATGCGATGC | 92-138 | 23 |
| (KU588278) |  | R:GATGACACTGCCGAAAC |  |  |
| *Eo*-1-77 | (AC)_12_ | F:[TAMRA]-TTCCGTGTGTAATGGTATC | 191-231 | 18 |
| (KU588287) |  | R:CCAATGTTGTTTGCGAC |  |  |
